# Diagnostic Accuracy of PCR in *gambiense* Sleeping Sickness Diagnosis, Staging and Post-Treatment Follow-Up: A 2-year Longitudinal Study

**DOI:** 10.1371/journal.pntd.0000972

**Published:** 2011-02-22

**Authors:** Stijn Deborggraeve, Veerle Lejon, Rosine Ali Ekangu, Dieudonné Mumba Ngoyi, Patient Pati Pyana, Médard Ilunga, Jean Pierre Mulunda, Philippe Büscher

**Affiliations:** 1 Department of Parasitology, Institute of Tropical Medicine, Antwerp, Belgium; 2 Rega Institute, Catholic University of Leuven, Leuven, Belgium; 3 Department of Parasitology, Institut National de Recherche Biomédicale, Kinshasa, Democratic Republic of the Congo; 4 Programme National de Lutte contre la Trypanosomiase Humaine Africaine, Mbuji-Mayi, Democratic Republic of the Congo; Yale School of Public Health, United States of America

## Abstract

**Background:**

The polymerase chain reaction (PCR) has been proposed for diagnosis, staging and post-treatment follow-up of sleeping sickness but no large-scale clinical evaluations of its diagnostic accuracy have taken place yet.

**Methodology/Principal Findings:**

An 18S ribosomal RNA gene targeting PCR was performed on blood and cerebrospinal fluid (CSF) of 360 *T. brucei gambiense* sleeping sickness patients and on blood of 129 endemic controls from the Democratic Republic of Congo. Sensitivity and specificity (with 95% confidence intervals) of PCR for diagnosis, disease staging and treatment failure over 2 years follow-up post-treatment were determined. Reference standard tests were trypanosome detection for diagnosis and trypanosome detection and/or increased white blood cell concentration in CSF for staging and detection of treatment failure. PCR on blood showed a sensitivity of 88.4% (84.4–92.5%) and a specificity of 99.2% (97.7–100%) for diagnosis, while for disease staging the sensitivity and specificity of PCR on cerebrospinal fluid were 88.4% (84.8–91.9%) and 82.9% (71.2–94.6%), respectively. During follow-up after treatment, PCR on blood had low sensitivity to detect treatment failure. In cerebrospinal fluid, PCR positivity vanished slowly and was observed until the end of the 2 year follow-up in around 20% of successfully treated patients.

**Conclusions/Significance:**

For *T.b. gambiense* sleeping sickness diagnosis and staging, PCR performed better than, or similar to, the current parasite detection techniques but it cannot be used for post-treatment follow-up. Continued PCR positivity in one out of five cured patients points to persistence of living or dead parasites or their DNA after successful treatment and may necessitate the revision of some paradigms about the pathophysiology of sleeping sickness.

## Author Summary

Post-treatment follow-up is crucial for sleeping sickness patient management and still relies on microscopic examination of the cerebrospinal fluid (CSF). Detection of the parasites DNA with the polymerase chain reaction (PCR) is proposed as a promising and possibly non-invasive alternative for monitoring treatment outcome, but has never been evaluated. We performed PCR on blood and CSF of 360 *Trypanosoma brucei gambiense* sleeping sickness patients, before treatment and during 2 years after treatment, and on blood of 129 controls. We found that performance of PCR to diagnose sleeping sickness and detect brain involvement was better or similar to current diagnostic techniques. However, we observed that PCR was unreliable for monitoring treatment outcome. Continued PCR positivity in cured patients points to persistence of parasites, or their DNA, after successful treatment, challenging the dogma that in sleeping sickness cure equals parasite elimination. In conclusion, we do not recommend PCR for treatment outcome assessment in sleeping sickness.

## Introduction

Human African trypanosomiasis (HAT) or sleeping sickness is caused by *Trypanosoma brucei* (*T. b.*) *gambiense* or *T. b. rhodesiense* and transmitted by tsetse flies. The disease is endemic in several countries in sub-Saharan Africa and usually found in remote rural areas. The Democratic Republic of the Congo (DRC) has the highest prevalence of *T. b. gambiense* HAT, with 8,000 to 25,000 cases diagnosed annually between 1997 and 2006 [1]. In the absence of prophylactic drugs or vaccines, disease control relies heavily on accurate diagnosis and effective treatment of patients. Diagnosis of *T. b. gambiense* HAT is generally based on serological screening of individuals followed by confirmation through parasite detection in lymph node aspirates or blood [2]. The disease progresses over two stages, the haemolymphatic (first stage) and the meningo-encephalitic phase (second stage), which require different drug regimens [3]. Hence, accurate staging of the disease is important for the therapeutic decision and done by microscopic analysis of the cerebrospinal fluid (CSF) [4]. The World Health Organization recommends a follow-up after treatment of 2 years before confirmation of definite cure, which is defined as the absence of trypanosomes, normalized white blood cell count in CSF and normalization of clinical signs [5].

In the past two decades, a wide range of different polymerase chain reaction (PCR) tests has been developed for trypanosome detection and identification [6]. PCR has frequently been presented as promising in HAT patient diagnosis, staging and post-treatment follow-up. However, very few PCR approaches have been evaluated on a large number of clinical specimens and none has been evaluated for detection of treatment failure in a prospective longitudinal study. Here we present the results of PCR on a series of 360 *T. b. gambiense* HAT patients who were enrolled in a 2-year prospective cohort. We evaluate the diagnostic accuracy of PCR in diagnosis, staging and detection of treatment failure.

## Methods

### Ethics statement

Samples from HAT patients were collected within a prospective observational study (THARSAT) [7]. The Commission for Medical Ethics of Institute of Tropical Medicine, Antwerp, Belgium and the Ethical Commission of the Ministry of Public Health, DRC approved this study. Negative control samples were collected within the TRYLEIDIAG study, which was approved by the commission for Medical Ethics of the University of Antwerp, Belgium and the Ethical Commission of the Ministry of Health DRC. All participants had given their written informed consent before inclusion into the study.

### Study participants

Human African trypanosomiasis patients were prospectively enrolled in the hospital of Mbuji Mayi, Kasai Province, DRC as described earlier [7] using the following inclusion criteria: trypanosomes in lymph node aspirate, blood or CSF; ≥12 years old; and living within a 100 km perimeter around Mbuji Mayi. Exclusion criteria were: pregnancy; no guarantee for follow-up; moribund condition; hemorrhagic CSF or; concurrent serious illness (tuberculosis, bacterial or cryptococcal meningitis). The cohort consisted of primary HAT cases (never treated previously for HAT) and retreatment HAT cases (previously treated for HAT, but with reappearance of trypanosomes in CSF at inclusion). All cases were treated according to the national guidelines [7]. First stage patients were treated with pentamidine, second stage cases with melarsoprol, eflornithine, or melarsoprol/nifurtimox, melarsoprol/eflornithine or eflornithine/nifurtimox combination therapy [7]. Patients were monitored for treatment outcome over 2 years.

Healthy endemic controls were recruited in the hospital of Mbuji-Mayi and at the University of Kinshasa [8]. A person was considered as a negative control when there was no clinical suspicion for HAT, no history of HAT, CATT was negative, and no trypanosomes were detected in blood.

### Reference standards

Sensitivity of PCR for HAT diagnosis was estimated in patients with a primary episode of HAT that was parasitologically confirmed by microscopic examination. Trypanosomes were detected by sequential examination of lymph node aspirate, blood examination by the capillary centrifugation technique or the mini-anion exchange centrifugation technique or examination of the CSF by modified single centrifugation [9]–[11].

The reference standard for staging of HAT was examination of the CSF on white blood cell (WBC) count and presence of trypanosomes by modified single centrifugation [10], [11]. Patients with 0–5 WBC/µl and no trypanosomes in CSF were classified in first stage, those with >5 WBC/µl and/or trypanosomes in CSF in second stage.

To determine HAT treatment outcome, examinations of CSF and/or blood of HAT patients were performed at 3, 6, 12, 18, and 24 months after treatment [12]. Treatment outcomes are described in detail elsewhere [7]. Briefly, the outcome was treatment failure, if during follow-up one of the following events occurred: 1° trypanosomes reappeared (relapse); 2° the CSF WBC increased by >30 cells/µl compared with the lowest previous count (probable relapse) or was higher than 20 cells/µl at 24 months (probable relapse); 3° occurrence of clinical signs requiring rescue treatment in the opinion of the physician in charge (probable relapse), or ; 4° death possibly related to HAT (interviews with family members revealed that there was no clinical improvement after HAT treatment and before the patient died). Cases that were lost to follow-up, died during treatment or died over the following 2 years from non-HAT related causes were considered without outcome and excluded. The treatment outcome was cure, if none of the above described events occurred.

All examinations were done by laboratory technicians experienced in HAT diagnosis.

### PCR

From HAT patients, blood and CSF specimens for PCR analysis were collected at time of diagnosis and at each follow-up visit. 200 µl of blood taken on heparin was mixed with an equal volume of AS1 storage buffer (Qiagen, Germany). One ml of CSF was centrifuged and the sediment was resuspended in 200 µl of AS1 buffer. Specimens were shipped at ambient temperature to the Institute of Tropical Medicine Antwerp, Belgium (ITMA), where DNA was extracted with the QIAamp DNA blood mini kit (Qiagen, Germany). Blood of the healthy endemic controls was collected, transported and extracted as described earlier [8]. DNA extracts were stored at −20°C and analysed with a PCR targeting a short sequence within the 18S ribosomal RNA gene of the *Trypanozoon*. The 25 µL reaction mixture contained 1× PCR buffer (Qiagen), 2.5 mM MgCl_2_ (Qiagen), 200 µM of each dNTP (Roche), 0.8 µM of sense primer M18S-II-F-Tb (5′-CGTAGTTGAACTGTGGGCCACGT-3′) (Sigma), 0.8 µM of antisense primer M18S-II-R-Tb (5′-ATGCATGACATGCGTGAAAGTGAG-3′) (Sigma), 2.5 µg acetylated bovine serum albumin (Promega), 0.5 unit of HotStar *Taq* polymerase (Qiagen) and 2.5 µl of specimen DNA. An initial denaturation step of 94°C for 15 minutes to activate the HotStar *Taq* polymerase was followed by 40 cycles of 94°C for 30 seconds, 60°C for 30 seconds and 72°C for 30 seconds and a final elongation step at 72°C for 5 minutes. Amplified products were analysed by electrophoresis in a 2% agarose gel (Eurogentec) and U.V. illuminated (Syngene) after ethidium bromide staining (Sigma). Measures to minimize the risk of false positive results due to PCR contamination were taken, including physical separation of pre- and post-PCR manipulations and inclusion of negative control samples during DNA extraction and PCR. The person who performed and interpreted the PCR results was a junior scientist experienced in PCR, who was blinded to the results of the reference standard tests.

The analytical sensitivity of the PCR was evaluated using 10-fold serial dilutions of *T. b. gambiense* procyclic parasites (strain MBA) in 180 µl naïve human blood (ranging from 10,000 to 1 parasites) mixed with 180 µL AS1 lysis buffer (Qiagen, Germany). The analytical specificity was assessed with purified DNA from *T. b. gambiense* and *T. b. rhodesiense* (at 1 ng DNA per test) and from *Plasmodium falciparum*, *Leishmania donovani*, *T. cruzi*, *Mycobacterium tuberculosis* and *Schistosoma mansoni* (50 ng DNA per test).

### Data analysis

STATA version 10 (Statacorp, USA) was used for data analysis setting the level of significance at 0.05. Sensitivities and specificities with 95% confidence intervals (CI) were calculated. Diagnosis of treatment failure during follow-up implied exclusion from the sensitivity calculations at subsequent time points. The Pearson's chi-square or Fisher exact test were performed to compare proportions in different groups.

## Results

### Analytical sensitivity and specificity of the PCR

The PCR detected 1 parasite in a 180 µl blood sample while non-spiked control blood samples remained negative. *T. b. gambiense* and *T. b. rhodesiense* DNA was detected at 1 ng per test while purified DNA from the non-target pathogens at 50 ng per test did not generate a positive PCR signal.

### HAT patients and healthy endemic controls

**Figure 1:**
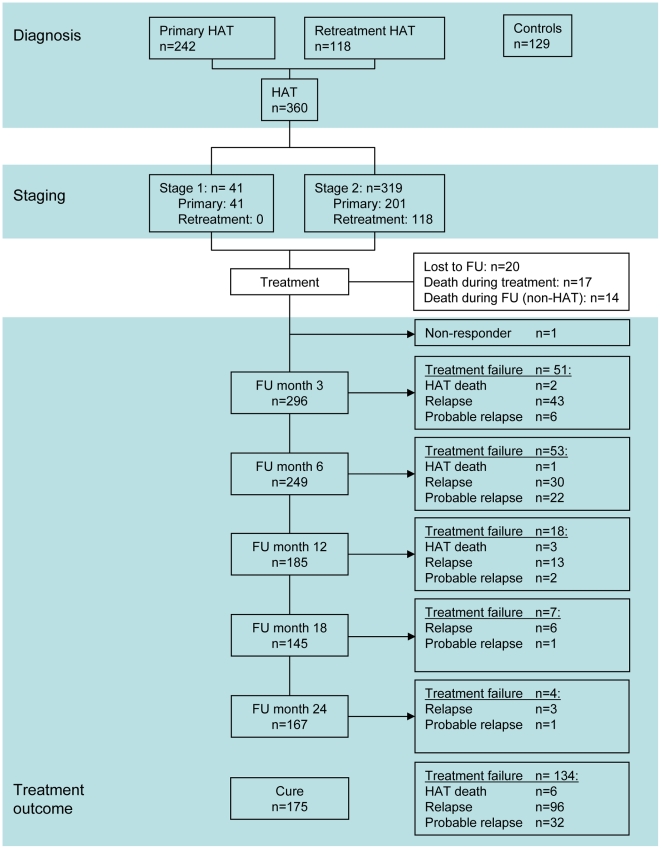
Classification of study participants according to reference standards for diagnosis, staging and follow-up (FU). At each follow-up time point, the number of patients attending is given. Patient groups reaching a final outcome of treatment failure during follow-up and excluded from analysis at subsequent time points, are indicated with an arrow.

A flow diagram of the study participants is presented in figure 1. Between January and September 2007, 129 healthy controls were recruited of which 23 were in Mbuji-Mayi and 106 in Kinshasa. Three hundred and sixty HAT patients were recruited between May 2005 and May 2008, of which 41 were in first stage and 319 in second stage. There were 242 primary episodes of HAT, and 118 patients presented with a recurrence of HAT characterised by trypanosome presence in CSF after unsuccessful previous treatment for HAT. Treatment outcome was cure in 175 HAT patients, while treatment failure occurred in 134. The number of patients presenting and the number and type of treatment failures diagnosed at each follow-up time point are shown in figure 1.

PCR analysis was performed between March 2006 and July 2009.

### PCR on blood for diagnosis of HAT

Specificity of PCR on blood (PCR-blood) was 99.2% and the sensitivity on primary HAT cases was 88.4% (table 1). The sensitivity was not significantly different between primary stage 1 and 2 patients (*p* = 0.184), but was significantly lower in retreatment cases (*p*<0.001).

**Table 1:** Sensitivity and specificity of PCR on blood for diagnosis of HAT.

	Blood			
Study cohort	Number of specimens	Number of positive PCRs	Sensitivity % (95% CI)	Specificity % (95% CI)
Control persons	129	1		99.2 (97.7–100)
All HAT patients	358	245	68.4 (63.6–73.3)	
Primary HAT	242	214	88.4 (84.4–92.5)	
Retreatment HAT	116	31	26.7 (18.6–34.8)	

Note. CI = confidence interval.

### PCR on CSF for staging of HAT

The specificity of PCR on CSF (PCR-CSF) for disease staging was 82.9% while the sensitivity was 88.4% (table 2). We did not observe a difference in sensitivity of PCR-CSF between primary and retreatment second stage patients (*p* = 0.559).

**Table 2:** Sensitivity and specificity of PCR on CSF for staging of HAT.

	CSF			
Study cohort	Number of specimens	Number of positive PCRs	Sensitivity % (95% CI)	Specificity % (95% CI)
1^st^ stage HAT	41	7		82.9 (71.2–94.6)
2^nd^ stage HAT	318	281	88.4 (84.8–91.9)	
Primary 2^nd^ stage	201	176	87.6 (83.0–92.2)	
Retreatment 2^nd^ stage	117	105	89.7 (84.2–95.3)	

Note. CI = confidence interval.

### PCR on blood for follow-up of HAT

In cured patients (table 3), the specificity of PCR-blood was 80.5% 3 months post-treatment, remained stable at a level of 89.9–92.4% between 6 and 18 months post-treatment to slightly decrease to 81.2% 24 months post-treatment. Between 12 and 24 months after treatment, 24.6% (95% CI 18.1–31.0) of cured patients had at least 1 PCR-blood positive result.

**Table 3:** Specificity and sensitivity of PCR on blood during post-treatment follow-up.

	Blood					
	Cured patients			Patients with treatment failure		
FU time point	Number of specimens	Number of positive PCRs	Specificity % (95% CI)	Number of specimens	Number of positive PCRs	Sensitivity % (95% CI)
Pre-treatment	174	101	42.0 (34.6–49.3)	134	109	81.3 (74.7–88.0)
3 months	169	33	80.5 (74.5–86.3)	126	27	21.4 (14.2–28.7)
6 months	168	17	89.9 (85.3–94.5)	80	10	12.5 (5.2–19.8)
12 months	157	12	92.4 (88.2–96.6)	27	8	29.6 (12.0–47.3)
18 months	132	10	92.4 (87.9–97.0)	10	2	20.0 (0–46.4)
24 months	160	30	81.2 (75.1–87.4)	4	2	50 (0.0–1.0)

Note. CI = confidence interval.

In patients that experienced treatment failure (table 3), the sensitivity of PCR-blood was at a low level of 12.5% at 6 months post-treatment and fluctuated between 20.0 and 50.0% later on.

### PCR on CSF for follow-up of HAT

In cured patients (table 4), the specificity of PCR-CSF slowly increased from 56.3% at 3 months after treatment to stagnate between 79.7 and 83.7% from 12 months post-treatment until the end of follow-up. Between 12 and 24 months post treatment, 35.4% (95% CI 28.3–42.6) of cured patients were CSF PCR positive on at least one occasion when tested.

**Table 4:** Specificity and sensitivity of PCR on CSF during post-treatment follow-up.

	CSF					
	Cured patients			Patients with treatment failure		
FU time point	Number of specimens	Number of positive PCRs	Specificity % (95% CI)	Number of specimens	Number of positive PCRs	Sensitivity % (95% CI)
Pre-treatment	174	126	27.6 (20.9–34.3)	134	119	88.8 (83.4–94.2)
3 months	167	73	56.3 (48.7–63.9)	126	91	72.2 (64.3–80.1)
6 months	168	63	62.5 (55.1–69.9)	80	55	68.8 (58.5–79.0)
12 months	157	27	82.8 (76.8–88.8)	27	17	63.0 (44.3–81.6)
18 months	129	21	83.7 (77.3–90.2)	10	4	40.0 (7.7–72.3)
24 months	158	32	79.7 (73.4–86.1)	4	2	50 (0.0–100.0)

Note. CI = confidence interval.

In patients that experienced treatment failure (table 4), the sensitivity of PCR-CSF decreased from 72.2% at 3 months post-treatment to 50% at the end of follow-up.

## Discussion

In this study, PCR applied to blood for diagnosis of primary *T.b. gambiense* HAT had 88% sensitivity and excellent specificity. For staging, sensitivity of PCR on CSF was 88%, specificity 83%. Sensitivity of PCR for detection of treatment failure during follow-up was low in blood. In successfully treated patients, parasite DNA disappeared only slowly from CSF, and remained detectable, albeit intermittently, in around 20% of the cured patients until the end of follow-up.

This is the first study in which PCR was performed systematically over the complete 2 years post-treatment follow-up of a large cohort of well characterised *T.b. gambiense* sleeping sickness patients with known treatment outcome.

Several considerations need to be considered in evaluating the data. For example, the patients enrolled in the cohort were a mixture of primary and retreatment cases so the sensitivity of PCR at diagnosis was estimated in primary cases only. Since patient enrolment in the study required that parasites had been detected by microscopic examination the sensitivity of PCR for HAT diagnosis might be overestimated given that patients with relatively high parasitemia were more likely to be included. Moreover, patients were treated with 6 different treatment schedules [7] and this may influence duration of PCR positivity after treatment, although no obvious differences were observed between groups (data not shown).

Follow-up examinations in the cohort focused on CSF examination, and once trypanosomes had been detected in CSF of relapsing patients, further blood examination was not carried out for some patients. Most treatment failures were detected within 12 months after treatment, which decreased the power of the study for reliable estimations of PCR sensitivity for treatment failure later on. We opted to use primers targeting the 18S ribosomal RNA gene, a multi-copy gene, offering high sensitivity given the relatively low analytical sensitivity of current *T.b. gambiense* specific PCR [13]. The M18S-II-Tb RNA gene primers will detect DNA from other members of the *Trypanozoon* group, including the closely related, but not pathogenic, *T.b. brucei*. Specificity of the primer set was tested on purified DNA from non-target pathogens and on blood of a control group, including endemic control samples collected in the same laboratory in Mbuji-Mayi as the HAT samples. A positive result in one of the uninfected control subjects originating from Mbuji-Mayi might be attributable to DNA from non-human infectious *T. b. brucei* in a tsetse bite. Since overall DNA yield after extraction from the blood samples was not recorded, negative PCR results due to unsuccessful DNA extraction cannot be excluded.

Specificity of PCR could not be tested on CSF of non-HAT endemic controls given the ethical considerations in collecting CSF from uninfected individuals.

For diagnosis of primary HAT, PCR on blood seems to show a comparable sensitivity and similar high specificity as the *Trypanozoon* OligoC-TesT targeting the same gene [8]. As such, PCR performance compares favourably to the best parasite detection techniques in current practice [14]. Given the technical requirements for PCR, the approach cannot yet be recommended for HAT patient diagnosis in the field [6]. However, it definitely has potential in travel medicine clinics, as an alternative or complement to the laborious microscopic techniques, and most likely, is also interesting for epidemiological research.

PCR on CSF also has some potential for disease staging. The observed sensitivity of CSF PCR is slightly lower than the 96–100% observed earlier [15]–[17]. However, these studies applied less sensitive trypanosome detection techniques and a primer set (TBR 1–2) that might be more sensitive than the M18S-II-Tb primers targeting the ribosomal RNA genes. Failure to diagnose second stage HAT may have serious consequences for patients, as most live in remote areas and might not get a second chance for proper diagnosis and treatment. We observed positive PCR results in CSF of some first stage patients, as has been reported previously [16], [17], apparently compromising specificity. Although if the shortcomings of the actual reference standard for staging are taken into account [4], the PCR approach might actually be demonstrating better staging sensitivity than the classical staging techniques [16]. The CSF-PCR positive first stage patients, with one exception, were however successfully treated with pentamidine, confirming earlier findings [17]. Thus, positive PCR results in otherwise normal CSF are to be interpreted with care.

The DNA detected in CSF could originate from parasites killed in the blood, either by crossing the blood brain barrier or by contamination during lumbar puncture, or from parasites that died in the CSF, taking into account the unfavourable survival conditions in that body fluid [18]. It could also originate from trypanosomes that invaded the CSF without CNS injury or inflammation and that are subsequently cleared with first stage drugs [16], [17]. The latter hypothesis is supported by reports of sleeping sickness patients with normal white blood cell counts, but with trypanosomes in CSF, that have been successfully treated with pentamidine [19], [20]. Interestingly, out of 11 second stage patients with white blood cell counts between 6 and 20/µL and no parasites observed in CSF, only 3 were positive in CSF-PCR (data not shown). Although the sample number is too low to draw major conclusions, this area of ambiguity, with few PCR positives, confirms the limitations of using a cut-off of 5 white blood cells per µL to make a diagnosis of stage 2 disease [4].

For patient follow-up after treatment, PCR on blood was neither sensitive nor specific. Poor sensitivity of PCR on blood was observed in the patients that were included in the study as retreatment cases, and was confirmed during follow-up. Previous studies [21] also indicated that PCR on blood is not an appropriate technique for detection of treatment failure in *T. brucei* infections. These observations further support the hypothesis that relapses originate from the central nervous system [22], and that trypanosomes are not likely to be found in blood of relapsing patients [23]. Lack of specificity , i.e. detectable parasite DNA in blood of some cured patients up to 24 months after treatment, corroborates earlier observations in *T.b. gambiense* infected mice where DNA was detected for a significant time period beyond successful melarsoprol treatment [24]. Although we cannot exclude the possibility that PCR-blood positivity among cured patients is caused by tsetse bites contaminated with non-human pathogenic *T.b. brucei* [25], this seems unlikely given the high diagnostic specificity on the uninfected control subjects.

PCR on CSF to determine treatment outcome seems to be of limited diagnostic value. Indeed, sensitivity of PCR on CSF for treatment failure varied from 40 to 72% and the phenomenon of PCR positive results in cured patients occurred even more frequently than in blood. Two case reports on CSF-PCR positivity after cure from *T.b. gambiense* infection have been published. Two CSF samples taken at 4 and 14 months after eflornithine treatment from a patient who was eventually cured, were positive in PCR with TBR 1–2 primers [26]. Using the same primers, two cured second stage patients out of a series of 15, were PCR positive in CSF immediately and 1 month after treatment [15], [27]. The reason for persistent CSF-PCR positivity of some apparently cured HAT patients remains obscure. Although not indicated by the internal PCR controls, false positive test results due to carry-over contamination can never be fully excluded. It may be also due to DNA from killed trypanosomes stabilised in the form of immune complexes [28] or in other ways and thus remaining detectable for a long period even when the patient is cured. This is in contrast with the finding that in herpes simplex viral encephalitis DNA detected by nested PCR disappears from CSF within 20 days after initiation of successful treatment [29]. Integration of *T.b. gambiense* DNA sequences into the host genome is another possibility. For example, spliced leader associated conserved DNA sequences of *T.b. brucei* have been documented in hepatocytes and kidney tubule cells of pentamidine treated, parasite negative, but intermittently PCR positive rats [30]. Furthermore, kinetoplast minicircle DNA from *T. cruzi*, a related kinetoplast causing American trypanosomiasis, has been shown to integrate into the human genome [31]. PCR positive results during follow-up might also point to the presence of living trypanosomes, i.e. continued brain infection, although patients were declared cured from the disease 2 years after treatment. This might underline a possible need to differentiate between parasitological and clinical cure. Indeed, some *T.b. gambiense* isolates do induce silent infections in the brains of mice that survived without clinical signs and without detectable parasites for more than 12 months, but with fluctuating PCR positivity [32]. In the same way, low amounts of DNA that fluctuate above and below the PCR detection limit may underlie the observed intermittent positivity in PCR results in the patient cohort studied here. Persistent silent *T.b. gambiense* infection even with treatment, could be explained by parasites residing in pharmacologically privileged sites (for example within host cells or in organs or tissues with reduced drug accessibility). Using *in vitro* models, the presence of *T.b. brucei* within astrocytes and entry of *T.b. gambiense* into microvascular endothelial cells of the human blood-brain barrier have been claimed [33], [34]. Persistence of *T.b. brucei* in the testis has also been shown in a mouse model after suboptimal drug dosage [35].

Our findings have important clinical implications for patient follow-up after treatment. While new algorithms based on normalised white blood cell count and the absence of trypanosomes in CSF may allow for cessation of follow-up at 6 months for the majority of patients [7], PCR on CSF remains positive in a significant fraction of the cured patients. A role of PCR or other DNA detection methods for improved post-treatment follow-up therefore is questionable. Fearing the repeated lumbar punctures during follow-up, most patients stay away from post-treatment controls [23], [36], [37]. PCR on blood has been proposed as a non-invasive alternative to replace the invasive CSF examination, but this is not supported by our results.

Confirmation of the results on other patient cohorts using other PCR primer sets or other DNA detection methods such as loop mediated isothermal amplification would be desirable [38]. If persistence of DNA or parasites in clinically cured patients is confirmed, it will be valuable to verify with quantitative real-time PCR if the DNA concentration in CSF correlates to treatment outcome. Clearly more work will be needed to fully understand the biological mechanism of DNA or parasite persistence and the dogma that cure equals parasite elimination may need revision.
